# Allele-selective von Willebrand factor silencing

**DOI:** 10.1016/j.rpth.2026.103366

**Published:** 2026-01-28

**Authors:** Jeroen Eikenboom, Noa Linthorst, Yvonne Jongejan

**Affiliations:** Department of Internal Medicine, Division of Thrombosis and Hemostasis, Leiden University Medical Center, Leiden, The Netherlands

**Keywords:** RNA interference, small interfering RNA, thrombosis, von Willebrand disease, von Willebrand factor

## Abstract

A State-of-the-Art lecture entitled “Allele-selective von Willebrand Factor (VWF) silencing” was presented at the International Society on Thrombosis and Haemostasis (ISTH) congress in 2025. The concept, potential applications, and feasibility of allele-selective *VWF* inhibition will be discussed in detail in this review. VWF plays a crucial role in supporting hemostasis, which is important in bleeding as well as thrombotic disorders. Decreased or functionally defective VWF results in von Willebrand disease (VWD), whereas high VWF levels have been associated with thrombotic risk. By silencing the synthesis of VWF from the mutant *VWF* gene in VWD, one might eliminate the production of mutant VWF and thereby normalize multimer composition and increase the levels of functional VWF. This would lead to phenotypic improvement in VWD. In the context of high plasma VWF levels and thrombotic disorders, silencing of one *VWF* allele will lower VWF in the circulation and endothelial cells, but at the same time, allele-selective silencing prevents an excessive reduction in VWF levels. Limited VWF reduction will reduce thrombosis risk without inducing bleeding. Selectively silencing the expression of one *VWF* allele, based on a single nucleotide difference between the 2 alleles, using small interfering RNAs has proven to be successful. This approach resulted in phenotypic improvement for VWD *in vitro* as well as *in vivo*. Preliminary data in the context of thrombotic risk indicated that silencing one *VWF* allele reduced thrombosis development without increasing bleeding. Finally, we summarize relevant new data on other new treatments for VWD presented during the 2025 ISTH Congress.

## Essentials

• von Willebrand factor (VWF) plays a pathophysiological role in bleeding and thrombosis.
• Customized small interfering RNAs enable selective silencing of a single *VWF* allele.
• Inhibiting expression of dominant-negative *VWF* variants is feasible and may improve VWF activity.
• Allele-selective *VWF* silencing may also safely reduce VWF levels in thrombotic disorders.

## 1 Introduction

von Willebrand factor (VWF) is a multidomain, multimeric protein synthesized in endothelial cells and megakaryocytes, stored in endothelial cells and platelets, and circulates in the bloodstream after secretion from endothelial cells. VWF has specific functional domains for binding to collagen as well as glycoprotein receptors on platelets [1,2]. As such, VWF plays a crucial role in the adhesion of platelets to exposed collagen at sites of vascular damage and in the aggregation of platelets, supporting platelet-platelet interactions. Via its mode of action, VWF is important in the pathophysiology of bleeding as well as thrombotic disorders. When VWF is deficient or functionally defective, reduced VWF platelet binding activity will result in an increased risk of bleeding and ultimately in von Willebrand disease (VWD). VWD is the most common inherited bleeding disorder and is divided into several (sub)types depending on the pathophysiology and severity of the disease (types 1, 2A, 2B, 2M, 2N, and 3) [[1], [2], [3]]. Contrary to low VWF, increased VWF levels and activity have been associated with increased risk for thrombotic disorders, such as myocardial infarction, ischemic stroke, venous thromboembolism, and thrombotic thrombocytopenic purpura [[4], [5], [6], [7], [8]]. Current treatment modalities for VWD have several shortcomings, and treatment for thrombotic disorders related to high VWF levels is usually not specifically targeted at VWF.

In VWD, treatment with 1-8-deamino-D-arginine vasopressin (DDAVP), a synthetic analog of vasopressin, induces secretion of endogenous VWF from the storage organelles, Weibel-Palade bodies, in the endothelium. Whether this treatment is effective depends on how much VWF can be secreted and its half-life in circulation and on the intrinsic functional defect of VWF, as it is the mutant VWF that will be secreted. In many cases, the increase in VWF after DDAVP treatment is too limited or of short duration only, and in some cases, the use of DDAVP is contraindicated, as the secreted mutant VWF itself may have deleterious effects, such as thrombocytopenia in type 2B VWD. Furthermore, frequent administration of DDAVP is required because its action is short-lived and the effect fades after repeated use (tachyphylaxis). When DDAVP cannot be used, the alternative is VWF concentrate, either plasma-derived or recombinant. VWF concentrates also require repeated intravenous infusions, are costly, and may induce the formation of inhibitory antibodies, especially in type 3 VWD. Finally, not all products have optimal multimeric composition, and some concentrates may lead to rather high factor VIII levels upon repeated administration. In both cases, with DDAVP as well as VWF concentrates, the mutant VWF remains in the circulation and that may have negative effects by itself, such as thrombocytopenia in type 2B VWD and potential development of intestinal angiodysplasia as seen in many cases of type 2 VWD [9,10].

Increased thrombotic risk associated with high VWF activity is usually treated with antiplatelet drugs, which inhibit the aggregation of platelets but are specifically targeted to platelets and do not interact with VWF pathways [11]. These platelet inhibitors may induce the risk of bleeding and do not always prevent the (re)occurrence of thrombosis [11]. For thrombotic thrombocytopenic purpura, the specific VWF-targeted therapy, caplacizumab, is a monoclonal antibody directed against the VWF-A1 domain and thereby prevents the interaction between ultra-large VWF multimers and glycoprotein Ib receptors on platelets, and consequently inhibit platelet adhesion [12]. An increased bleeding risk, leading to frequent bleeding symptoms, is inherent to this functional blockade of platelet adhesion [12].

An innovative treatment for VWD would eliminate the mutant VWF protein from the circulation, normalize the multimer composition, increase the levels of functional VWF, and allow for long treatment intervals. In the context of high plasma VWF levels and thrombotic disorders, an ideal therapeutic option would be to reduce VWF in the circulation and endothelial cells and at the same time, to limit the reduction of VWF to levels at which it would not induce bleeding. Selective inhibition of only one of the *VWF* alleles could be such a new treatment modality for both sides of the coin, bleeding in VWD as well as thrombotic risk. The feasibility of allele-selective inhibition will be discussed in detail in this review.

## 2 The Concept of Allele-Selective *VWF* Silencing

**Figure 1:**
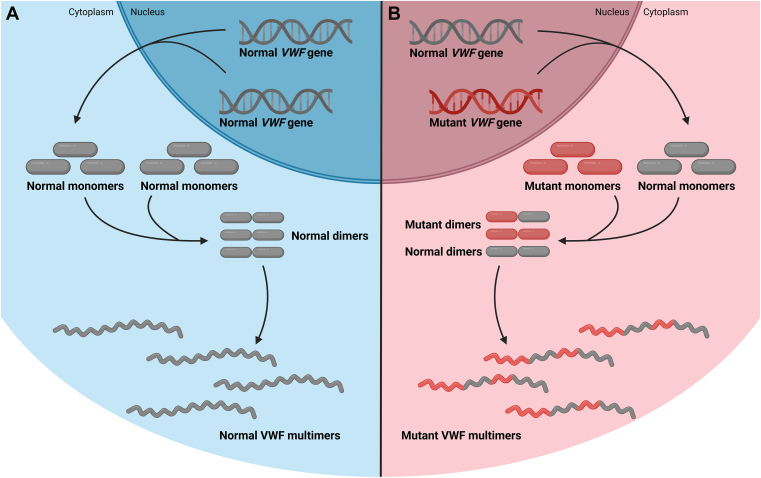
von Willebrand factor (VWF) synthesis. Schematic and simplified representation of VWF synthesis and posttranslational modifications. VWF is synthesized in endothelial cells and megakaryocytes as monomeric subunits that dimerize in the endoplasmic reticulum. These dimers subsequently form VWF multimers in the Golgi. (A) In the normal, healthy situation, both copies of the *VWF* gene code normal VWF subunits that form normal VWF dimers and normal VWF multimers. (B) When 1 of the 2 *VWF* alleles contains a missense mutation, normal as well as mutant VWF monomers are synthesized. During posttranslational modifications, mutant and normal monomers will be incorporated in the VWF dimers and multimers. Even though only one copy of the *VWF* gene is mutated, the resulting multimer will be composed of both mutant and normal VWF subunits. Hence, the mutation exerts a dominant-negative effect on the entire multimer.

VWF is synthesized in endothelial cells and megakaryocytes as a propolypeptide consisting of a signal peptide, a propeptide, and several structural domains. In the endoplasmic reticulum, the signal peptide is cleaved off, and the pro-VWF subunits dimerize via disulfide bridges at the carboxyterminal cystine knot. The resulting pro-VWF dimers move to the Golgi where VWF multimers are formed through disulfide bonds between the D'D3-domains of pro-VWF dimers. At this stage, the propeptide is cleaved off, resulting in mature VWF multimers. Via these posttranslational modifications, the VWF multimers are composed of VWF subunits that originate from both *VWF* alleles (Figure 1A) [1,2].

In VWD, one of the *VWF* alleles produces mutant VWF (eg, a single nucleotide missense mutation resulting in an amino acid change), and then the mutant as well as normal VWF subunits are both incorporated into the VWF multimer, thereby disrupting the entire composition of the multimer. The mutant VWF subunits exert a dominant-negative effect on the normal VWF subunits from the wild-type allele, and thus the entire VWF multimer is compromised (Figure 1B) [1]. This may lead to functionally defective VWF multimers, a lack of storage or intracellular retention of VWF, or a very short half-life of VWF after secretion. In fact, such a dominant-negative mutation has a more detrimental effect on VWF functionality than a mutation that creates a null allele with no protein production. When a null allele is present, only normal VWF subunits are produced by the other allele, and these form normal multimers, albeit at a somewhat lower concentration. Inhibiting the production of VWF only from the mutant allele would conceptually improve multimer composition and ultimately the VWD phenotype. Allele-selective *VWF* inhibition in VWD could be applied in patients heterozygous for dominant-negative *VWF* mutations (which is true in most cases of type 1 and in types 2A, 2B, and 2M VWD) and would improve a severe phenotype to an asymptomatic or very mild status, comparable to that of carriers of a *VWF* null allele. Allele-selective *VWF* inhibition would not be applicable in recessively inherited forms of VWD (types 2N and 3).

In patients with thrombotic disorders, there are no causative mutations in either of the *VWF* alleles, so for this indication the goal would not be silencing of a specific allele, but simply reduction of circulating VWF levels in general. The level of VWF reduction should be sufficient for reducing thrombosis risk while not inducing a bleeding phenotype. Allele-selective *VWF* inhibition in this case might be the solution, as it could limit the reduction of VWF levels to roughly 50%, enough to reduce thrombosis risk and safely prevent bleeding risk.

Thus, allele-selective *VWF* silencing might be a therapeutic option in VWD as well as thrombotic disorders in which VWF may play a pathophysiological role. How can this allele-selective inhibition be achieved?

## 3 Allele-Selective *VWF* Silencing *in Vitro*

**Figure 2:**
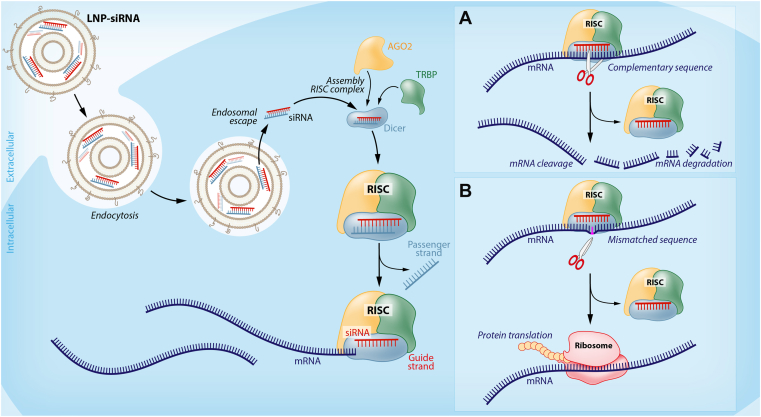
Small interfering RNA (siRNA)-mediated allele-selective silencing. Lipid nanoparticle (LNP)-siRNA complexes are taken up by the cell via endocytosis. After release into the cytoplasm through endosomal escape, the siRNA interacts with Argonaute-2 (AGO2), transactivation responsive RNA binding protein (TRBP), and Dicer to form the RNA-induced silencing complex (RISC). The passenger strand is cleaved off by RISC, which allows the guide strand of the siRNA to bind to the target mRNA. (A) When the siRNA is fully complementary to the target mRNA, RISC cleaves the mRNA, leading to mRNA degradation and inhibition of protein translation. (B) A mismatch between the guide strand of the siRNA and the target mRNA (for example, due to a heterozygous variant in one allele) will result in no cleavage of the mRNA by RISC, and the mRNA will be translated to protein.

A potential approach to silence the expression of a single allele is the use of RNA interference, a mechanism of posttranscriptional gene silencing mediated by small RNAs [13]. Synthetically produced double-stranded small interfering RNAs (siRNAs) can incorporate into the RNA-induced silencing complex, where the guide strand binds to the complementary target mRNA, leading to mRNA cleavage and degradation and thereby silencing of gene expression (Figure 2).

Several siRNA-based therapies have already entered the market, such as patisiran for treatment of hereditary transthyretin-mediated amyloidosis with polyneuropathy and inclisiran for hypercholesterolemia. Fitusiran, which inhibits the synthesis of antithrombin for the treatment of hemophilia A and B, has recently been approved by the US Food and Drug Administration [[14], [15], [16]]. In all these examples, the siRNA silences expression from both alleles of the targeted genes. However, for the application we envision for silencing mutant *VWF* expression, as outlined above, it is necessary to specifically silence only one of the *VWF* alleles. There are already examples of preclinical development of allele-selective silencing in autosomal dominant-negative diseases by applying siRNA as well as antisense oligonucleotides [[17], [18], [19], [20], [21], [22], [23]]. For VWD, Casari et al. [24] described partial *in vitro* correction of the dominant-negative effect in a patient with type 2A VWD carrying an in-frame *VWF* deletion using an siRNA targeting the mRNA deletion breakpoint. Subsequently, they also showed in an *in vivo* mouse model of type 2A VWD that siRNAs targeting the deletion breakpoint can limit the negative effects of the dominant-negative *VWF* allele, leading to partial phenotypic improvement in the VWF multimer profiles [25].

Based on these data, we have set out to develop a therapeutic siRNA strategy to silence only one *VWF* allele. To allele-selectively silence only one *VWF* allele, the siRNA should be targeted against a region where the coding sequence of both *VWF* alleles differs, and only the allele with full sequence complementarity between the siRNA and mRNA will be inhibited. In VWD, one could use the *VWF* mutation as the target for the siRNA; however, with hundreds of unique mutations causing VWD, it is not feasible to design an siRNA for each specific mutation [26]. Moreover, when applying this allele-selective approach in the context of thrombosis, there are no mutations in the *VWF* gene to be targeted. Therefore, we have chosen to direct the siRNAs against common genetic variants in *VWF* (single nucleotide polymorphisms, SNPs) with high minor allele frequency (Table) [27]. When an individual is heterozygous for a SNP, that SNP can be used as a target for the siRNA. In VWD, the targeted *VWF* SNP-allele should be located on the same chromosome, in *cis*, with the causative *VWF* mutation. However, either of the 2 SNP-alleles may be targeted in the context of lowering VWF levels in prothrombotic disorders, and thus even in the absence of *VWF* mutations, a SNP-based allele-selective siRNA inhibition is feasible. A similar SNP-based siRNA targeting approach has been described for treating patients with Huntington’s disease [28,29].

**Table:** SNPs in *VWF* selected as siRNA targets [27].

SNP	*VWF* cDNA location	Minor allele	Minor allele frequency	% Heterozygous
rs1800378	c.1451G|A	A	0.35	45.3
rs1063856	c.2365A|G	G	0.37	46.5
rs1063857	c.2385T|C	C	0.37	46.5
rs1800380	c.2880G|A	A	0.26	38.1

SNP, single nucleotide polymorphism; VWF, von Willebrand factor.

The feasibility of designing siRNAs that can selectively inhibit only one *VWF* allele based on a single nucleotide difference between the 2 alleles has been shown for 4 different *VWF* SNPs (Table) with high predicted heterozygosity [27]. Several siRNAs targeting the respective alleles of the 4 SNPs were tested, and 11 siRNAs were identified that showed potent dose-dependent and allele-selective inhibition of the targeted allele. *In vitro* cotransfections of *VWF* alleles with the most efficient of the siRNAs resulted in a remaining fraction of only 5% of the targeted allele in the entire sample, which could be enough inhibition to result in a phenotypic correction of dominant-negative mutations. So, siRNA-based allele-selective silencing depending on a single nucleotide mismatch seems feasible, but can this indeed mediate a phenotypic improvement?

## 4 Improvement of VWD Phenotype *in Vitro*

The first *in vitro* proof of improvement of the VWD phenotype based on allele-selective silencing was obtained for a type 2A VWD mutation [27]. The c.8318G>C (p.Cys2773Ser) mutation has been described to result in a type 2A phenotype, previously indicated as type 2A(IID), which is characterized by a distinct multimer electrophoresis pattern, with a lack of high-molecular-weight multimers and the presence of odd-numbered multimers in between the regular multimer bands [30]. This very specific abnormal multimer pattern can be accurately reproduced in human embryonic kidney (HEK)293 cells by transient cotransfection with plasmids encoding wild-type *VWF* and p.Cys2773Ser mutant *VWF*. The potential correction of the phenotype by siRNA-based allele-selective silencing was investigated in these cotransfected cells using siRNAs targeting the *VWF* SNP-allele located on the same construct as the variant coding the p.Cys2773Ser mutant *VWF*. The VWF multimer pattern showed improvement for most siRNAs tested, and this was quantified by an increase in the VWF large multimer index [27]. However, combining siRNAs targeting different SNPs to improve efficacy did not result in further improvement of the effect.

Although the cotransfections in HEK293 cells can molecularly mimic the heterozygous VWD phenotype, the potential overexpression of the cDNA constructs in this system may not properly reflect human *in vivo* expression of the 2 *VWF* alleles. Also, there is no guarantee that cotransfected cells express both constructs equally. Therefore, a potentially more physiologically relevant cell model would be endothelial colony-forming cells (ECFCs), which can be isolated from peripheral blood of the patient [31,32]. In these patient-derived ECFCs, both *VWF* alleles are naturally expressed. ECFCs isolated from a patient with type 2A VWD caused by a heterozygous dominant-negative *VWF* variant (c.3569G>A, p.Cys1190Tyr) were studied [33]. The ECFCs from the patient heterozygous for the p.Cys1190Tyr mutation were characterized by VWF with reduced high-molecular-weight multimers, reduced binding of VWF to collagen, intracellular retention of VWF in the endoplasmic reticulum, and an increase in unprocessed pro-VWF in the lysates of the ECFCs. To apply the SNP-based siRNA approach to these patient-derived ECFCs, heterozygosity for one of the candidate SNPs is required. The patient was heterozygous for the *VWF* SNP c.1451A|G, and the 1451A variant was determined to be located on the same allele as the mutant c.3569A (p.Cys1190Tyr). Treatment of the ECFCs with an siRNA targeted against the SNP-allele 1451A resulted in an allele-selective decrease in mRNA expression of the targeted allele as measured by allele-selective quantitative polymerase chain reaction. At the protein level, this resulted in an improvement of the multimer pattern in the culture medium, an increase in collagen binding activity in cell lysate, and a decrease in unprocessed pro-VWF in cell lysate, whereas when the normal *VWF* allele was inhibited by the complementary siRNA targeted against the 1451G allele, all phenotypic parameters dramatically deteriorated. The intracellular retention of unprocessed VWF in the endoplasmic reticulum of the patient-derived ECFCs was clearly visualized via immunofluorescence staining. Treatment with the siRNA against the mutant 1451A allele resulted in a clear decrease of endoplasmic reticulum retention, and many cells no longer showed VWF retention. However, inhibition with siRNA against the normal 1451G allele led to a severe cellular phenotype with major endoplasmic reticulum retention of VWF [33]. The strongest inhibitory effect of the siRNA-based *VWF* silencing in ECFCs was observed 6 days after siRNA treatment, and only after 28 days did *VWF* expression return to the level of untreated cells [33]. Similar *in vitro* phenotypic correction has been shown in ECFCs from a patient with another heterozygous type 2A VWD mutation (c.3568T>C; p.Cys1190Arg) after allele-selective knockout of the mutant *VWF* allele using CRISPR/Cas9 targeting of the same *VWF* SNP c.1451A|G [34].

These studies delivered important proof of concept of the feasibility, efficacy, and selectivity of siRNA-mediated as well as CRISPR/Cas9-based allele-selective silencing of *VWF* targeting SNP-alleles instead of the causative *VWF* mutation. However, can these promising *in vitro* data be confirmed *in vivo*?

## 5 Allele-Selective *VWF* Silencing *in Vivo*

siRNA-based silencing is sequence-specific, and human *VWF* sequence-based siRNAs, as described in the previous paragraphs, cannot be assessed *in vivo* in any existing animal model. Therefore, animal model-specific siRNAs are required. Furthermore, application of *VWF*-silencing siRNAs *in vivo* requires endothelial-specific delivery vehicles. Finally, an allele-selective approach targeting SNP-alleles is not possible in mice, as inbred mouse strains do not have SNPs and are fully homozygous for all genetic loci. An approach to mimic heterozygous SNPs in human *VWF* is by using the genetic differences between various mouse strains. When crossing 2 different mouse strains, the first filial generation (F1) of offspring will be heterozygous for all genetic differences between their genes. Following this principle, 2 mouse strains were selected, C57BL/6J and 129S1/SvImJ, that have 11 nucleotide differences between the coding sequences of their respective *Vwf* genes. As a proxy for the human SNP-approach, several siRNAs were designed to target those specific strain-selective differences [35]. For delivery of the siRNAs to the endothelium *in vivo,* the siRNAs were encapsulated in 7C1 oligomeric lipid nanoparticles (LNPs) as developed by Dahlman et al. [36].

A *Vwf*-silencing siRNA encapsulated in the 7C1-LNP was very effectively targeted to the endothelium in C57BL/6J and 129S1/SvImJ mice, resulting in a dose-dependent inhibition of both *Vwf* mRNA (isolated from lung) and plasma VWF protein [35]. For one of the strain-selective siRNAs targeted against the C57BL/6J *Vwf* sequence, the inhibition of plasma VWF was up to 90% in homozygous C57BL/6J mice. The degree of inhibition was still the same after 10 days. Immunofluorescent images of the lungs of C57BL/6J mice showed nearly complete inhibition of VWF staining in the endothelium. Testing the allele selectivity of the strain-selective siRNAs in a heterozygous setting, the siRNAs were administered to the F1 hybrids from a cross between C57BL/6J and 129S1/SvImJ mice [37]. Treatment with the strain-selective siRNAs, either selective for the C57BL/6J or the 129S1/SvImJ mouse *Vwf* sequence, resulted in an approximately 50% reduction in lung *Vwf* mRNA as well as plasma VWF in the F1 hybrids, which is expected when only one of the *Vwf* alleles is effectively and selectively inhibited.

Thus, it can be concluded that endothelial delivery of siRNAs *in vivo* is feasible, and that effective allele-selective inhibition can be obtained. However, can this proof of principle of allele-selective silencing of VWF translate into meaningful phenotypic modulation?

## 6 Impact of Allele-Selective Silencing Of VWF *In Vivo*

The therapeutic potential of allele-selective siRNAs to correct a VWD phenotype *in vivo* has been studied in a murine model of type 2B VWD. This mouse model harbors the c.3946G>A (p.Val1316Met) type 2B VWD mutation, which naturally occurs in VWD patients [38]. As the murine type 2B VWD model has been created in a C57BL/6J mouse background, a similar approach could be followed by creating heterozygous F1 hybrids by crossing the type 2B VWD C57BL/6J mice with wild-type 129S1/SvImJ mice, resulting in heterozygous type 2B VWD mice with the mutation on the C57BL/6J *Vwf* allele. Treating those heterozygous type 2B VWD mice with the siRNA selectively directed against the C57BL/6J *Vwf* sequence resulted in selective inhibition of the mutant (c.3946G>A; p.Val1316Met) *Vwf* allele [39]. This treatment reduced total VWF by approximately 50%, and this reduction was shown by targeted quantitative protein mass spectrometry to be mainly selective inhibition of the mutant allele. The selective inhibition of the mutant allele improved the VWD type 2B phenotype as reflected by a normalization of the plasma VWF multimers, an increase in VWF collagen binding, and a decrease in the number of platelet aggregates in peripheral blood smears. The bleeding phenotype was tested by a tail-clip bleeding assay and showed a correction of the bleeding time in 4 of 6 mice compared with a prolonged bleeding time in all 6 untreated mice [39]. These results support the potential applicability of allele-selective silencing of mutant dominant-negative *VWF* alleles as a new therapeutic approach for VWD.

As discussed in the introduction, the allele-selective silencing of VWF may also be applied outside the context of VWD as a therapeutic strategy to inhibit VWF synthesis and thereby reduce thrombotic risk. This has also been investigated [37]. Reducing VWF plasma levels using allele-selective siRNAs in the F1 hybrids from a cross between wild-type C57BL/6J and 129S1/SvImJ mice resulted in approximately 50% reduction of plasma VWF, as anticipated. This controlled reduction in VWF did not prolong tail-bleeding time in those mice, indicating that a 50% reduction of VWF is safe with regard to bleeding risk. The effect of this allele-selective silencing of *Vwf* on thrombus development was investigated in a FeCl_3_-induced vascular injury model [37]. When analyzing the occurrence of occlusive thrombi in mesenteric arterioles and venules, no difference was observed between treated and untreated mice; however, the presence of nonocclusive thrombi was significantly reduced in mesenteric arterioles upon treatment of the F1 mice with the allele-selective siRNA. If a *VWF-*silencing siRNA is to be applied in a proatherothrombotic setting, adequate delivery to the endothelium of an atherosclerotic vasculature is required. This has been studied in proatherothrombotic *APOE∗3-Leiden*.CETP mice (homozygous C57BL/6J background) [40]. It was shown that the siRNA targeting the C57BL/6J *Vwf* sequence could be efficiently delivered to the endothelium in mice with hypercholesterolemia and advanced atherosclerosis, which resulted in a strong reduction in plasma and endothelial VWF. Although the thromboprotection of this siRNA application has not been studied extensively, these first studies support further development of this potential therapeutic approach.

## 7 Summary and Future Perspectives

**Figure 3:**
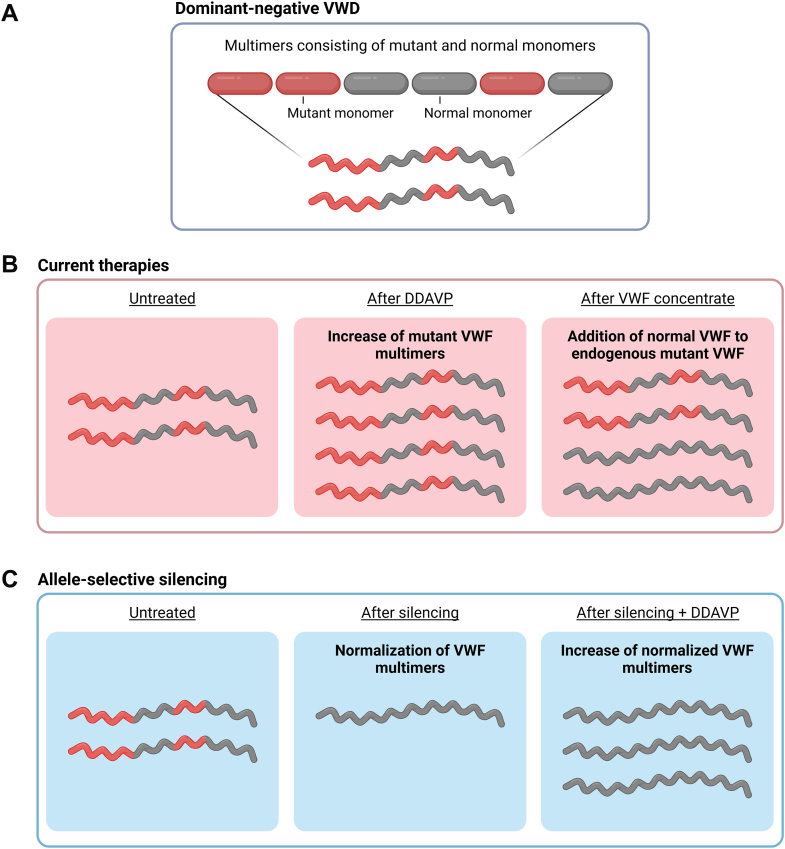
Current treatment vs allele-selective silencing in dominant-negative VWD. (A) In VWD caused by dominant-negative mutations, the VWF multimers are composed of mutant as well as normal VWF subunits, causing all circulating VWF to be abnormal. (B) Current therapies for VWD do not remove the mutant VWF from the circulation. During treatment with DDAVP, the level of endogenous VWF may rise, but this will all be mutant VWF. Treatment with VWF concentrates adds normal VWF to the circulation; however, the endogenous mutant VWF remains present and may still have negative phenotypic effects (eg, thrombocytopenia in type 2B VWD, development of intestinal angiodysplasia). (C) With allele-selective silencing, the production of mutant VWF subunits is inhibited, resulting in improved VWF multimers composed of normal subunits only. This will lead to an increase in VWF activity despite a reduction in the concentration of total circulating VWF. After allele-selective silencing of VWF, additional treatment with DDAVP will result in induced secretion of normal VWF. DDAVP, 1-8-deamino-D-arginine vasopressin (desmopressin); VWD, von Willebrand disease; VWF, von Willebrand factor.

As shown by the studies outlined above, allele-selective *VWF* silencing based on a single nucleotide allelic difference is possible using siRNA- as well as CRISPR/Cas9-based approaches. Targeting the siRNAs to endothelial cells *in vivo* with major selective knockdown of the targeted allele is feasible when the siRNAs are encapsulated into LNPs. Proof of concept of phenotypic correction upon allele-selective silencing has been shown for different VWD-causing mutations: *in vitro* in HEK293 cells with siRNA treatment for the type 2A mutation c.8318G>C (p.Cys2773Ser); e*x vivo* in patient-derived ECFCs with siRNA treatment for the type 2A mutation c.3569G>A (p.Cys1190Tyr); e*x vivo* in patient-derived ECFCs with CRISPR/Cas9 treatment for the type 2A mutation (c.3568T>C; p.Cys1190Arg); and *in vivo* in C57BL/6J mice with siRNA treatment for the type 2B mutation (c.3946G>A; p.Val1316Met). Based on these promising results, allele-selective silencing could be a new treatment modality for VWD caused by heterozygous dominant-negative *VWF* mutations (including most type 1 and types 2A, 2B, and 2M VWD). Application of allele-selective silencing in VWD will require sequencing of the *VWF* gene to identify the causative variant as well as to determine heterozygosity of the targetable SNPs and which SNP variant is in *cis* with the *VWF* mutation. The approach of allele-selective silencing is unique in the sense that it is the only treatment that actually removes the mutant VWF from circulation (Figure 3). Currently, it is unclear how often patients would require redosing of siRNAs for allele-selective silencing. siRNA-based silencing *in vitro* in human ECFCs showed that expression of VWF had returned to the level of untreated cells after 28 days [33]. In *in vivo* mouse studies, siRNA-based silencing resulted in dose-dependent inhibition of up to 90% of plasma VWF, and the degree of inhibition remained the same at the RNA level 10 days after treatment [35]. The effect of the siRNA silencing is therefore expected to be prolonged. The siRNAs used in the above mentioned experiments are not the ones that will be used for clinical application. Clinically applicable siRNAs are currently being developed and may have even longer duration of activity. So, frequency of redosing patients is not yet known, but redosing intervals of at least several weeks are envisioned. The experimental administration of siRNA in mice was intravenous; however, a subcutaneous administration would be preferred. Recently, new therapeutic strategies for VWD have gained interest, and other developments for treatment of VWD were presented at the ISTH congress in 2025. The monovalent antibody HMB-002 elevates the levels of endogenous circulating VWF by prolonging the half-life of VWF, which mainly limits its application to type 1 VWD [[41], [42], [43]]. A novel gene therapy approach for VWD was also presented, which is based on AAV-mediated VWF propeptide delivery and which can potentially correct VWF multimerization and storage defects [44]. However, this approach is restricted to mutations located in the VWF propeptide region.

Allele-selective *VWF* silencing could have a wider application than VWD only. As a therapeutic approach in thrombotic conditions, preliminary data in mice suggest that allele-selective silencing of *VWF* does not result in increased tail-bleeding time and may reduce thrombus formation in a FeCl_3_-induced vascular injury model. However, allele-selective silencing of *VWF* could be a therapeutic modality for any disorder in which increased VWF levels or activity play a pathophysiological role. Apart from atherothrombotic conditions, one could envision treatment of thrombotic thrombocytopenic purpura [8], sickle cell disease in which high VWF levels contribute to vaso-occlusive episodes [45,46], liver cirrhosis in which elevated VWF levels are associated with progression of disease [47,48], or even cancer [49]. For these indications, the same siRNAs can be used as developed for VWD, but now either of the SNP-alleles may be targeted provided that the patient is heterozygous for the SNP. Considering all these options, further development of allele-selective silencing of *VWF* is warranted.

Future directions for developing allele-selective *VWF* silencing will include further testing of the phenotypic improvement in different VWD causing variants and in appropriate thrombotic models. The definitive therapeutic siRNA compounds need to be designed and validated. The siRNAs evaluated *in vivo* in mice were designed for complementarity to the mouse sequence and cannot be translated to the human situation. The siRNAs described in the *in vitro* phenotypic improvement studies were directed against the human *VWF* sequence; however, those siRNAs need to be optimized regarding efficacy as well as selectivity. This will require testing different siRNA sequences as well as introducing chemical modifications. When the optimal siRNA compounds have been identified, off-target effects need to be evaluated in endothelial cells and toxicity needs to be assessed in pharmacologically relevant cell types. Further preclinical testing will require the development of ‘humanized’ models as the human-specific siRNAs cannot currently be tested in any existing animal model due to lack of homology. Only at that stage can clinical trials be anticipated.
